# 

**Published:** 1889-10-05

**Authors:** 


SATURDAY, OCTOBER 5th, 1889.

**"London Empty"** - - - - - - - - - - 1

**Words of Consolation**.—Peace - - - - - - - 2

**The Doctor's Pulpit.**—"Below Par" - - - - - - 3

**The Story of the Insane from Year to Year.**—Asylum Reports—Summary - - - - - - 4

**The Hospital Workshop.** X.—In the Wards at Great Ormond-street Hospital for Children - - - 5

**Unqualified Assistants and Death Certificates.** By INDEX - - - - - - - - 6

**Everybody's Page** - - - - - - - - - 7

**Annotations.**— Progressive Sanitation in America — Mr. Tooth as a Hypnotist—A Scientific Delusion - - - 8

**Notes and News** - - - - - - - - 9

**The Practitioner's Outlook and Retrospect** - - 11

**German System of National Insurance** - - - 15

**Turning Over New Leaves.**—Algerian Hints for Tourists - - - - - - - - 16

**Scraps and Gleanings** - - - - - - - - 16

**EXTRA SUPPLEMENT—THE NURSING MIRROR.**

**En Passant.**—From Far Isles—Exeter Workhouse—Birkenhead Nurses' Institute—Short Items—Infirmary Nursing—Salisbury Infirmary Walk - - - - - i
**Case Taking.**—Second Paper - - - - - - - ii
**Answers to Examination Questions** - - - - ii
**Presentations—Lectures** - - - - - - - - iii
**An Exhibition of Nursing Uniforms.**—The Doll Competition - - - - - - - - - iii
**Everybody's Opinion.**—Examination Questions - - iv
**Amusements and Relaxation** - - - - - - iv
**Appointments—Vacancies—Notes and Queries** - iv
**Miss Valeria** - - - - - - - - - - v

